# Why Does Amphibian Chytrid (*Batrachochytrium dendrobatidis*) Not Occur Everywhere? An Exploratory Study in Missouri Ponds

**DOI:** 10.1371/journal.pone.0076035

**Published:** 2013-09-25

**Authors:** Alex Strauss, Kevin G. Smith

**Affiliations:** Tyson Research Center and Department of Biology, Washington University in St. Louis, St. Louis, Missouri, United States of America; University of California Riverside, United States of America

## Abstract

The amphibian chytrid fungus, *Batrachochytrium dendrobatidis* (Bd), is a globally emerging pathogen that has caused widespread amphibian population declines, extirpations, and extinctions. However, Bd does not occur in all apparently suitable amphibian populations, even within regions where it is widespread, and it is often unclear why Bd occurs in some habitats but not others. In this study, we rigorously surveyed the amphibian and invertebrate biodiversity of 29 ponds in Missouri, screened resident amphibian larvae (*Rana (Lithobates*) sp.) for Bd infection, and characterized the aquatic physiochemical environment of each pond (temperature pH, conductivity, nitrogen, phosphorus, and chlorophyll-a). Our goal was to generate hypotheses toward answering the question, “Why does Bd not occur in all apparently suitable habitats?” Bd occurred in assayed amphibians in 11 of the 29 ponds in our study area (38% of ponds). We found no significant relationship between any single biotic or abiotic variable and presence of Bd. However, multivariate analyses (nonmetric multidimensional scaling and permutational tests of dispersion) revealed that ponds in which Bd occurred were a restricted subset of all ponds in terms of amphibian community structure, macroinvertebrate community structure, and pond physiochemistry. In other words, Bd ponds from 6 different conservation areas were more similar to each other than would be expected based on chance. The results of a structural equation model suggest that patterns in the occurrence of Bd among ponds are primarily attributable to variation in macroinvertebrate community structure. When combined with recent results showing that Bd can infect invertebrates as well as amphibians, we suggest that additional research should focus on the role played by non-amphibian biota in determining the presence, prevalence, and pathogenicity of Bd in amphibian populations.

## Introduction

In recent decades, emerging infectious diseases of wildlife have come to be recognized among the greatest threats to global biodiversity [1]–[3]. This is partially due to evidence of emerging pathogens causing significant declines across multiple taxa, including mammals [4], birds [5], and corals [6]. In particular, the amphibian chytrid fungus, *Batrachochytrium dendrobatidis* (Bd), is a potent threat to global amphibian biodiversity that has caused amphibian population declines, extirpations, and extinctions across several continents [7]–[9]. Like other declines caused by epizootics, Bd-associated declines can be severe and rapid [10]. As a result, research on Bd is often conducted with urgency and as a response to a crisis and therefore typically focuses on those locations where the pathogen is known both to occur and to cause amphibian mortality. This crisis-based approach has led to a rapid accumulation of knowledge about the causes and consequences of amphibian population declines caused by Bd [11].

Despite the benefits and necessity of addressing Bd-associated amphibian declines as a crisis and prioritizing research accordingly, many fundamental questions about the ecology of Bd still remain unanswered. Specifically, relatively little is known about the abiotic and biotic factors that limit where Bd occurs at fine spatial scales, perhaps because sites where Bd does not occur (or does not cause declines) are often ignored and are not studied. Thus, although broad predictions can be made about the potential geographic distribution of Bd based on its known distribution, [12], [13], relatively little is known about why Bd occurs where it does at a finer scale, for example among habitats or sites within a region (but see[14], [15]). This is an important consideration because while Bd is essentially omnipresent in some regions [9], [10], in others (e.g., parts of Africa, the United States, and Asia) Bd occurs only in a subset of apparently suitable sites, i.e., aquatic habitats with populations of amphibians [16]–[20].

Why does Bd not occur in all amphibian populations in a region? Few field studies directly address this question. Climatic factors such as temperature and precipitation are clearly important, based on empirical laboratory and field studies [12], [14], [21]. But these variables may be less relevant at small spatial scales and in similar habitat types, which often have similar climatic environments (but see [22]). Several community-level factors, including amphibian host density [23], host species identity [24], and diversity [25] have also been shown to be important to Bd prevalence in laboratory studies, but their contribution to patterns of distribution of Bd in natural habitats is unknown. Other biotic and abiotic variables that are important to the structure and diversity of aquatic habitats, including water chemistry, habitat isolation, zooplankton community structure, and macroinvertebrate community structure, have not been studied with respect to the occurrence of Bd in natural habitats. Moreover, it is not known how sensitive the occurrence of Bd might be to natural spatial variation in any of these properties; for instance, the turnover of species among communities, (i.e., beta diversity [26]).

We suggest that fully understanding Bd as an emerging pathogen requires broader exploratory research to understand among-site occupancy patterns of this pathogen and the community context in which Bd occurs. Studying the patterns of Bd occurrence in this ecological framework may provide valuable information on why Bd does or does not occur in particular habitats and can help generate hypotheses to guide research on possible locations of future emergence or possibly identify *in situ* refuges for vulnerable amphibian species.

Within this framework, we designed an exploratory field study of the occurrence of Bd in amphibian populations in pond habitats of east-central Missouri, USA. In this region, Bd occurs in approximately 30–50% of surveyed pond habitats occupied by amphibians, but has not been observed to cause mortality or declines (K.G. Smith, unpublished data). From 29 physically-similar ponds, we assayed larval amphibians for Bd, collected physical and chemical data, and conducted intensive aquatic biodiversity surveys. Our primary aim was to identify correlative patterns in Bd occurrence, especially relating to the biological communities of ponds, with the ultimate goal of generating novel explanatory hypotheses to explain occupancy patterns of Bd in natural amphibian populations. With this goal in mind, we first conduct some simple univariate analyses and then apply more sophisticated multivariate community ecology analyses (ordination and permutational multivariate analysis of variance) to ask, “Are there identifiable differences in the abiotic and biotic characteristics of ponds that correspond with presence or absence of Bd?” Finally, we test for evidence of direct and indirect effects of these multivariate relationships and develop causal hypotheses via structural equation modeling, and conduct a similarity percentage analysis in order to identify species that could be ecologically important for Bd.

## Methods

### Ethics statement

This research was approved by the Washington University Institutional Animal Studies Committee (protocol approval # 20070102). All field research was conducted in accordance with Missouri Department of Conservation guidelines under MDC Wildlife Collector's Permit #14048.

### Overview of Biology of Batrachochytrium dendrobatidis

Bd is an aquatic pathogen of amphibians that infects keratinizing tissue, including skin in adult amphibians and mouthparts in larval anurans, i.e., tadpoles [27]. Although Bd is a generalist pathogen, it does not infect all amphibian species equally ([28]–[30]). Until recently, Bd was not known to have alternative hosts or environmental reservoirs, but nematodes [31] and crayfish [32] have recently been documented as hosts of Bd. Bd is generally considered to be intolerant of temperatures above 28–32°C and desiccation [33]. Thus, while terrestrial amphibians can be infected with Bd, wetlands and streams are generally considered to be “hotspots” for Bd owing to high densities of host species and appropriate environmental conditions [34], [35]. It is for these reasons that we focus on understanding occurrence of Bd within aquatic habitats in Missouri.

### Statement on Sampling Design and Scientific Philosophy

We deliberately designed this study to be correlative and exploratory in nature. Our primary goal is to identify novel correlations in occurrence of Bd at the landscape scale to contribute to the development of new hypotheses, thereby expanding the scope of current research on Bd. As a result, the causative pathways discussed in this paper are presented with the goal of guiding future research.

Given our sampling and analytical design, which resulted in large amounts of data and multiple tests for associations among variables, it is possible that some of our significant relationships are spurious and arise as a result of random chance alone. For this reason we emphasize the results which have multiple independent forms of supporting evidence and conclude by reiterating that our results should be interpreted as hypotheses and not conclusions.

### Study region

**Figure 1:**
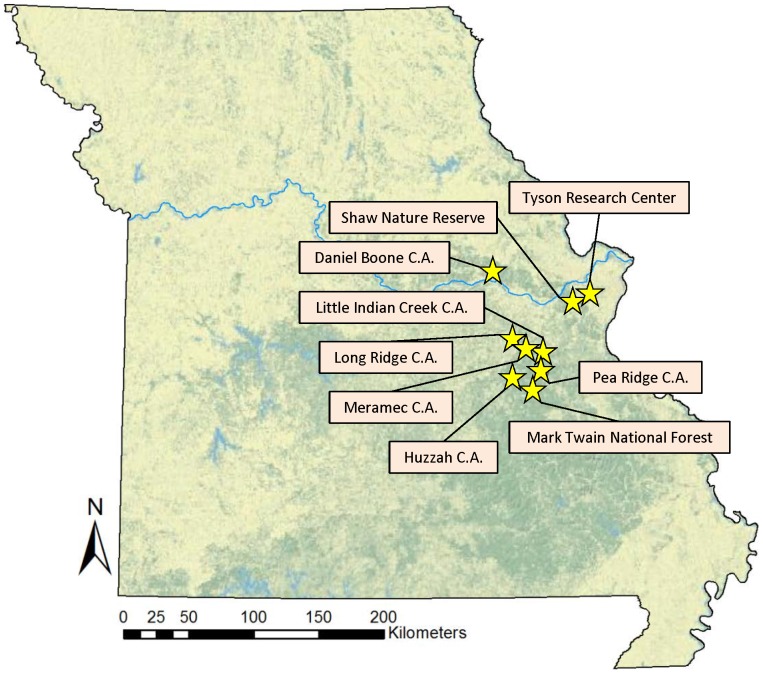
Map of locations of study sites within Missouri, USA.

We visited 29 ponds from nine natural areas in east-central Missouri during June and July, 2009 (figure 1). One pond was located at Long Ridge Conservation Area, two at the Missouri Botanical Garden's Shaw Nature Reserve, two at Little Indian Creek Conservation Area, three at Tyson Research Center of Washington University in St. Louis, four at Daniel Boone Conservation Area, three at Pea Ridge Conservation Area, four at Huzzah Conservation Area, five at Meramec Conservation Area, and five at Mark Twain National Forest near Potosi, Missouri. Together, these conservation areas span five counties in east-central Missouri. The average distance between conservation areas is 45 kilometers, and the average pairwise distance between ponds within a conservation area is 1.24 kilometers. All of the ponds are permanent, having held water year-round over the past five years, and contain amphibian populations but not fish. The ponds have similar dimensions (diameter of ∼20 meters and maximum depth of ∼1 meter), open canopies, rocky/muddy bottom compositions, and are embedded in an oak-hickory forest matrix.

During the study period, we documented the presence of nine amphibian species regionally, including Southern leopard frogs (*Rana (Lithobates) sphenocephala*), green frogs (Rana (*Lithobates) clamitans*), pickerel frogs (*Rana* (*Lithobates) palustris*), gray tree frogs (*Hyla versicolor/chrysoscelis*), spring peepers (*Pseudacris crucifer*), Blanchard's cricket frogs (*Acris crepitans*), American toads (*Bufo (Anaxyrus) americanus*), Central newts (*Notophthalmus viridescens*), and spotted salamanders (*Ambystoma maculatum*). Of these species, *R. sphenocephala*, *R. clamitans*, and *R. palustris* overwinter as larvae (tadpoles) in the study region, and *N. viridescens* overwinter as adults, making these species candidates as year-round Bd reservoirs[36]. We also identified 59 species of zooplankton and 80 species of macroinvertebrates, including 34 species of Coleoptera (aquatic beetles and beetle larvae), 18 species of Odonata (dragonfly and damselfly larvae), 12 species of Hemiptera (aquatic true bugs), 9 species of Gastropoda (snails), 5 species of Diptera (midge and mosquito larvae), and representatives from several other taxa.

### Bd assay

We assayed larvae of the three anuran species with long-lived larval stages (*Rana sp.*) for Bd because they are potential Bd reservoirs [36] and are often infected with Bd in this region (K.G. Smith, unpublished data). Approximately 30 tadpoles were collected from each pond using dip nets and euthanized on-site with MS-222. Their oral discs were excised with a scalpel, stored in centrifuge tubes filled with 70% ethanol [37], and assayed for the presence of Bd via quantitative PCR using positive and negative controls [38]. The blade of the scalpel was cleaned with alcohol wipes after every five tadpoles in order to remove Bd zoospores and minimize contamination between tadpoles. Inter-individual contamination was assessed via field controls, which were collected for each pond by washing the scalpel blade in a centrifuge tube of 70% ethanol once during tadpole processing at each pond. All field controls were negative for Bd, indicating that contamination among tadpoles did not occur (see Results). All field equipment was sprayed and saturated with dilute bleach solution between ponds in order to prevent spreading of Bd among study sites [39].

### Aquatic biodiversity and pond biotic and abiotic variables

While visiting each pond, we also characterized the general abiotic and biotic environment of each pond by collecting data on aquatic biodiversity and water chemistry. Since our ponds were geographically close and in equivalent landscapes, they experienced similar climatic and weather conditions, including seasonal rainfall and temperature regimes and therefore did not collect detailed weather data (see Fig. 1 for spatial scale of study sites). Instead, we measured variables that often influence the structure of aquatic ecological communities, even at relatively small spatial scales (i.e., among ponds within a natural area). Our abiotic variables were sampling day (to detect seasonal effects), water temperature at time of sampling, distance to nearest neighboring pond (to account for host dispersal ability), water conductivity and pH, and total nitrogen and phosphorus. We measured conductivity and pH onsite using a YSI multimeter, while total nitrogen and total phosphorus were determined in the laboratory using water samples collected from each pond and a portable spectrophotometer. Finally, we calculated the distance between each pond and its nearest neighboring pond for a metric of pond isolation, using arcGIS (ArcGIS Desktop: Release 10; ESRI, 2011).

For our biotic variables, we collected data on diversity and abundance of amphibian, macroinvertebrate, and zooplankton species in each pond. For estimates of relative species densities, a standard spatially-constrained sampling technique was used, in which three 36 cm×1 m tall plastic cylinders were inserted into the bottom of each pond and all organisms were removed from the enclosed water column with a dip net (“stovepipe” sampling; [40]). Ten additional sweeps with a dip net were used in the open water of each pond to catch fast-swimming or rare macroinvertebrate species. Species incidence indices incorporated data from both of these sampling methods while abundance indices used exclusively the stovepipe data. Amphibian species were identified in the field, counted, and released; macroinvertebrate species were collected and preserved in 70% ethanol and identified later with a dissecting microscope and standard field guides and keys. Zooplankton were sampled by filtering 4 liters of water through a plankton net at ten different locations in each pond; these samples were then preserved with Lugol's iodine solution and identified in the laboratory with a compound microscope and key. From each pond we also collected a water sample for quantification of chlorophyll-a concentrations using a portable fluorometer.

### Univariate analyses

We used logistic regressions to identify significant correlations between each of our biotic and abiotic metrics and presence or absence of Bd, and we used Fisher's exact tests to test for nonrandom co-occurrence patterns between Bd and each individual amphibian species. Finally, we also used a Mao tau test to rarefy and compare regional species richness (of amphibians, macroinvertebrates, and zooplankton) between Bd and non-Bd ponds, using PAST version 2.12 (Paleontological Statistics; Hammer, Harper, and Ryan, 2001).

### Multivariate ordinations

We used these same data to characterize the 29 surveyed ponds in multivariate ecological space. We used non-metric multidimensional scaling (NMDS, using PAST) to ordinate ponds in two ways. First, we ordinated ponds according to their species assemblages, using the Raup-Crick and Bray-Curtis similarity indices for amphibians, macroinvertebrates, and zooplankton. Second, we ordinated ponds according to their physiochemical properties using a Euclidean distance metric and standardized measurements of conductivity, pH, total nitrogen, total phosphorus, and chlorophyll-a. In these ordinations, ponds were classified according to presence or absence of Bd to identify qualitative differences in multivariate characters of the two groups of ponds.

### Multivariate tests of beta diversity (using PERMDISP2 and SEM)

We used permutational analysis of multivariate dispersions (PERMDISP2, Anderson, 2006) to test for differences in the multivariate dispersion distances (i.e., biotic and abiotic similarity) between ponds in which Bd did and did not occur. This test asks whether Bd ponds tend to be more (or less) similar to each other in biotic or abiotic characteristics than are non-Bd ponds. When ponds are ordinated by the Raup-Crick similarity index, dispersion distance is analogous to the concept of beta diversity, or species turnover [26], [41]; when ponds are ordinated by the Bray-Curtis metric, dispersion distance more broadly indicates variability in community structure as a function of turnover and species abundance. When ponds are ordinated by their water chemistry variables, dispersion distance is measure of chemical dissimilarity of the two groups of ponds.

Next, because our amphibian incidence, macroinvertebrate incidence, and physiochemical ordinations all showed that Bd only occurred in a restricted subset of all ponds based on these variables (see Results), we designed structural equation models (SEM) using Mplus (Mplus Version 6.1; Muthen & Muthen, 1998–2010) to identify direct and indirect links between variation in pond physiochemistry, amphibian and macroinvertebrate beta diversity, and the incidence of Bd between ponds. Structural equation models allow for the development and testing of hypothesized causal relationships in correlational data. The model included terms for the average species and physiochemical dissimilarities of each pond (as independent and dependent variables, respectively), which were calculated from the dissimilarity matrices used in the Raup-Crick and Euclidian distance ordinations. Bd incidence (presence/absence) was included as a binary dependent variable. The model was estimated with a robust weighted least squares estimator using a diagonal weight matrix. When the final model structure was determined, its fit was ensured with chi-square difference testing, in which the final model was nested within the saturated model.

### Identification of potentially relevant species (using SIMPER)

As a supplementary analysis, we identified the macroinvertebrate species whose abundances contributed most to the observed differences between Bd and non-Bd ponds. We accomplished this by conducting a similarity percentage (SIMPER) analysis [42] in PAST using the macroinvertebrate Bray-Curtis abundance-based index. Of the 68 macroinvertebrate species for which we had density estimates, we determined the species whose abundances were most skewed between the groups of Bd and non-Bd ponds, and report the species whose cumulative contributions explain 50% of the overall dissimilarity between groups.

## Results

### Bd in the study region

Across all 29 ponds, we detected Bd in 78 of 793 larval anurans (overall infection prevalence of 9.8%). Mean infection intensity of infected individuals was very low, with 3.9 zoospore equivalents detected per animal on average. Mean infection intensity was positively correlated with infection prevalence in these ponds (R^2^ = 0.8276; p<0.001). All field controls tested negative for Bd, confirming that our sampling methods prevented contamination among individual amphibians within a pond. No larvae had visibly deformed mouthparts and we found no evidence of Bd-related mortality in any amphibians at any of our sites. Overall, 11 of the 29 ponds contained larval *Rana* that tested positive for Bd. We detected no spatial structure among Bd and non-Bd ponds; ponds with Bd were found in six of the nine natural areas, and average within-group distances of Bd and non-Bd ponds was not distinguishable (average distance of Bd ponds: 40.4 km, average distance of non-Bd ponds: 44.6 km, p = 0.270). Twenty ponds contained *R. clamitans*, six contained *R. sphenocephala* and three contained *R. palustris*, but there was no significant relationship between the species that was tested for Bd and whether Bd was detected in a pond (Fisher's exact test, p>0.15).

### Univariate trends

We did not detect a significant correlation between Bd incidence and any of the biotic or abiotic variables that we measured (Tables S1 S2 and S3). Thus, we found no relationship between presence of Bd in a pond and sampling date, pond isolation, temperature, resident amphibian populations (total amphibian density, *Rana sp.* density, and *N. viridescens* density, local amphibian diversity, or individual species occupancies), diversity of other taxonomic groups (zooplankton density, zooplankton diversity, or macroinvertebrate diversity), or pond physiochemistry/productivity (conductivity, pH, total nitrogen, total phosphorus, and chlorophyll-a). We detected a trend that presence of Central Newts (*Notophthalmus viridescens*), one of the candidate reservoir species, was positively correlated with presence of Bd (Fisher's exact test; p = 0.058; Table S1). Greater amphibian and macroinvertebrate regional richness was found among non-Bd ponds (59 versus 52 macroinvertebrates species and 8 versus 7 amphibian species, after rarefaction).

### Multivariate ordinations and dispersion distances

**Figure 2:**
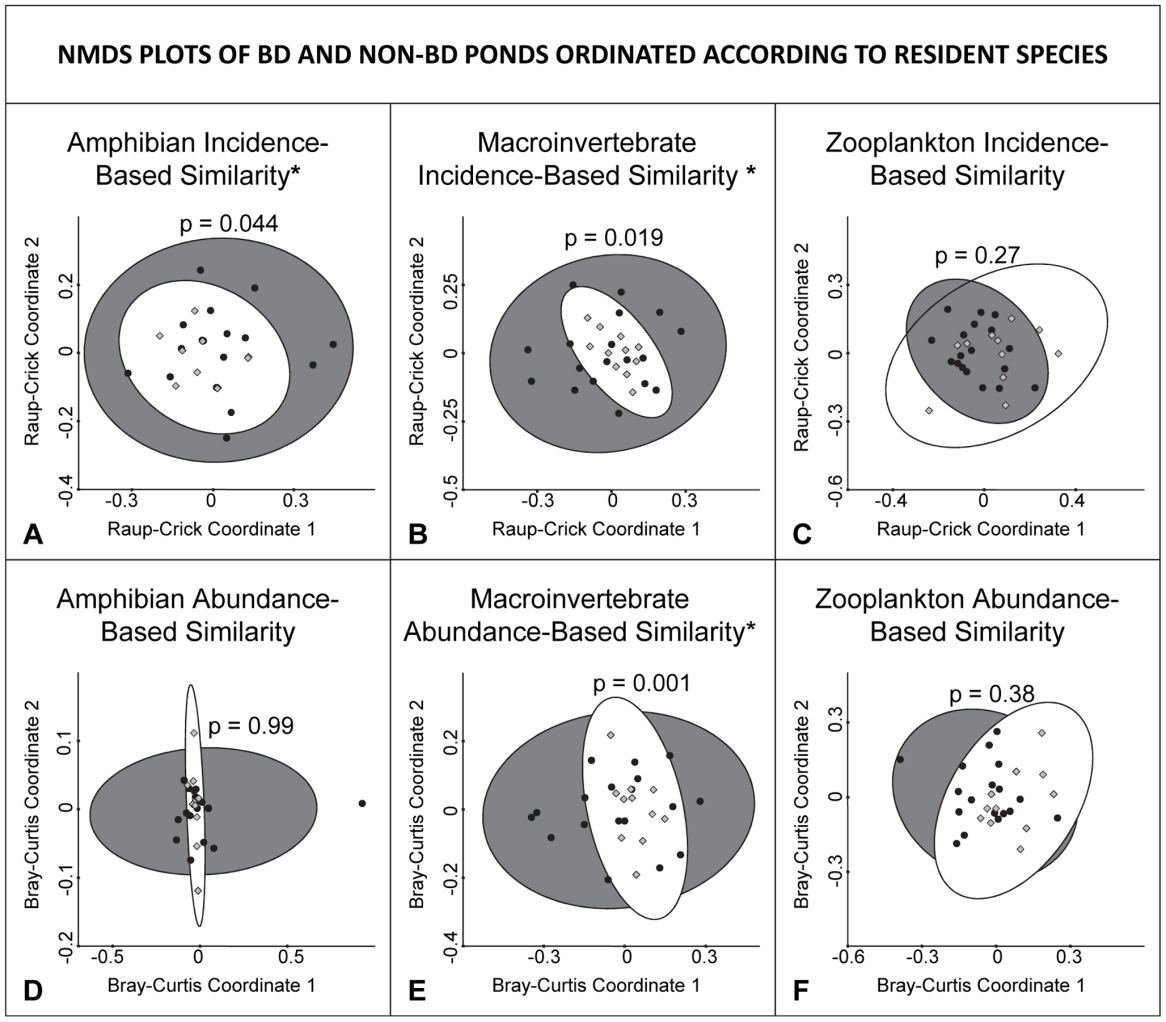
NMDS plots of Bd and non-Bd ponds according to their resident species. Ponds are ordinated according to Raup-Crick incidence-based indices (top row; A, B, and C) and Bray-Curtis abundance-based indices (bottom row; D, E, and F) of resident amphibian (left column; A and D), macroinvertebrate (center column; B and E), and zooplankton (right column; C and F) species. Bd ponds are light gray diamonds (encircled by the white 95% confidence ellipse) and non-Bd ponds are black circles (encircled by the dark 95% confidence ellipse). Ponds that are closer together are more similar to each other. In incidence-based ordinations (A, B, and C), dispersion distance is analogous to beta diversity. Dispersion difference is significantly lower (*) for groups of Bd ponds in A, B, and E.

Amphibian and macroinvertebrate communities, in terms of species incidences, were more similar among ponds with Bd than among ponds without Bd (Fig. 2A, amphibians, PERMDISP2 F = 4.48, p = 0.044 and Fig. 2B, macroinvertebrates, PERMDISP2 F = 6.25, p = 0.019). No significant difference was found for biotic similarity of zooplankton species incidences between groups (Fig. 2C). When ponds were ordinated according to the abundances of amphibians, macroinvertebrates, and zooplankton, the group of Bd ponds in the macroinvertebrate ordination again had a significantly lower dispersion distance (higher biotic similarity) than the group of non-Bd ponds (Fig. 1E, PERMDISP2 F = 12.83, p = 0.001). There was no significant difference in dispersion distances between Bd and non-Bd ponds for amphibian or zooplankton abundance-based ordinations (Figs. 2D and F).

**Figure 3:**
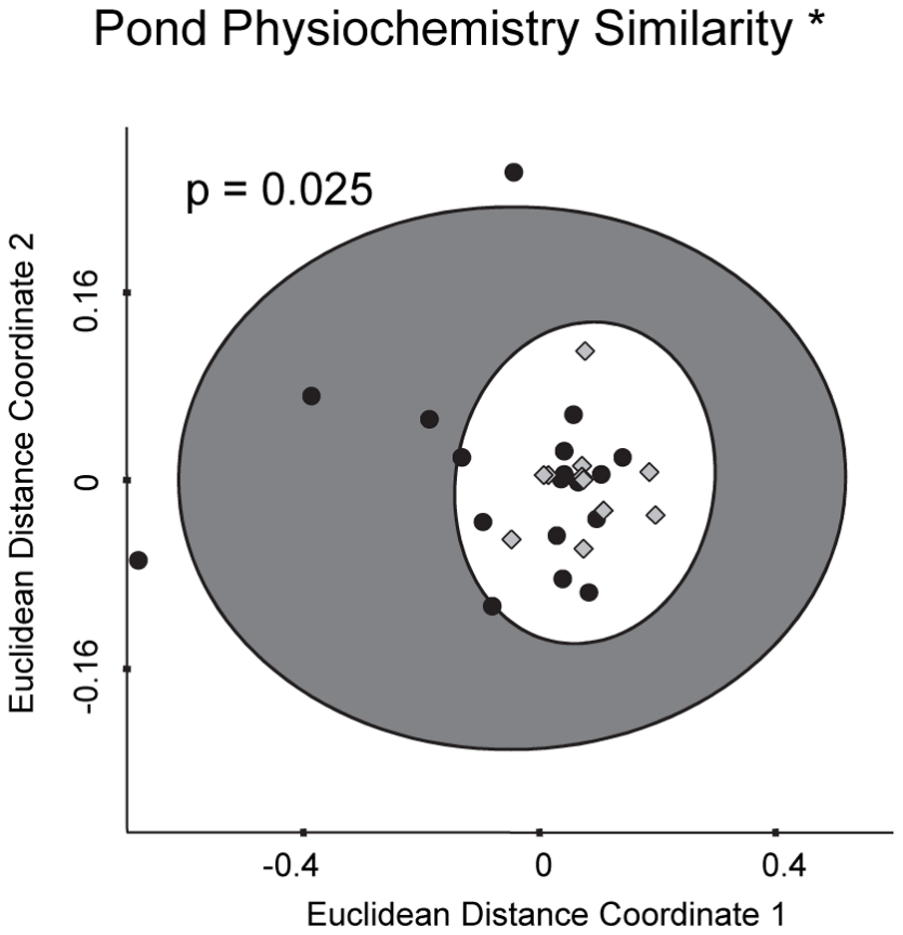
NMDS plot of Bd and non-Bd ponds according to pond physiochemistry. Ponds are ordinated according to Euclidian dissimilarities of standardized physiochemical variables (conductivity, pH, total nitrogen, total phosphorus, and chlorophyll-a). Bd ponds are light gray diamonds (encircled by the white 95% confidence ellipse) and non-Bd ponds are black circles (encircled by the dark 95% confidence ellipse). Ponds that are closer together are more similar to each other. Dispersion distance is significantly lower for the group of Bd ponds.

When ponds were ordinated according to their physiochemical characteristics, the group of Bd ponds also had a significantly lower dispersion distance than the group of non-Bd ponds (Fig. 3; PERMDISP2 F = 5.60, p = 0.025). In other words, Bd ponds were more similar to each other in terms of physiochemical properties than were non-Bd ponds.

### Structural equation modeling, biotic similarity, and Bd incidence

**Figure 4:**
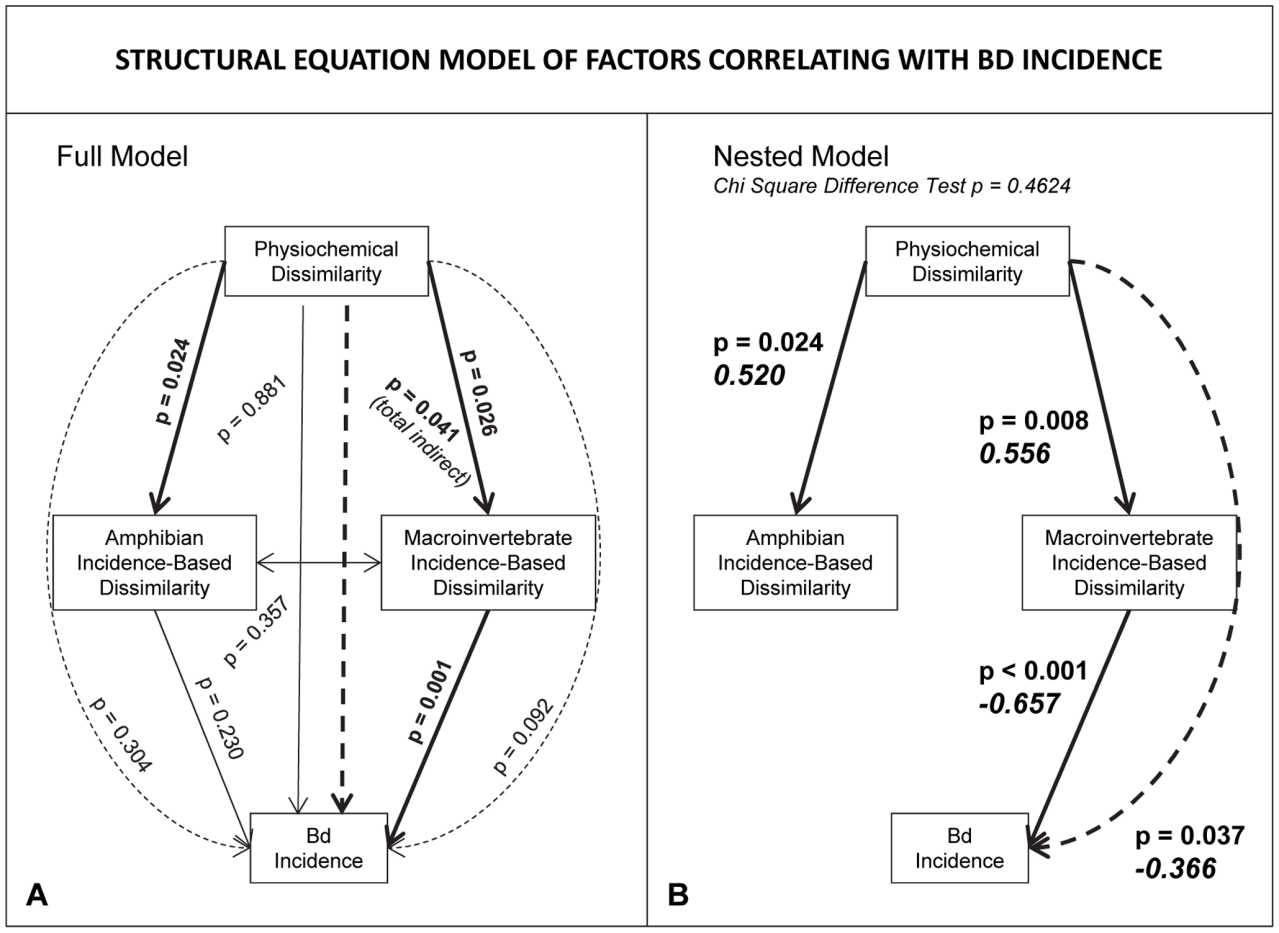
Structural equation modeling of factors correlating with Bd incidence. Structural equation modeling reveals that the correlation between physiochemical dissimilarity and Bd incidence is an indirect effect, mediated by macroinvertebrate community dissimilarity; macroinvertebrate community dissimilarity shares the only significant direct correlation with Bd incidence. Direct links are represented as solid lines and indirect links are represented as dashed lines. Significance of each correlation is reported as a p value, and significant links are emphasized in bold. Only significant links from the saturated model (A) were used to build the final, nested model (B). Standardized effect sizes for each link and a test of model fit are included (in italics) for the final model.

Significance of the correlations in the full (saturated) structural equation model (Fig. 4A) was used to inform construction of the final (nested) model (Fig. 4B). Chi square difference testing was used to evaluate the final model fit, relative to the full model it was nested within. The p value of 0.4624 indicates that the final model does not deviate significantly from the data and therefore the model cannot be rejected. Structural equation modeling suggested that the correlation between pond physiochemical dissimilarity and Bd incidence (see Fig. 3) is an indirect effect, mediated by macroinvertebrate community dissimilarity. Of the terms included in the model, only macroinvertebrate community dissimilarity shares a significant direct correlation with Bd incidence (as ordinated in Fig. 2B). This correlation is significant (p<0.001) and negative. The total indirect link between physiochemical similarity and Bd incidence was not included in the final model, because it was equivalent to the indirect link mediated by macroinvertebrate community dissimilarity. When the link between amphibian similarity and Bd incidence was included in the final model, it was nonsignificant and did not alter the significance of any other links.

### SIMPER analysis

Over 50% of the dissimilarity in macroinvertebrate communities between Bd and non-Bd ponds (Fig. 2E) was explained by abundances of 10 of the 68 macroinvertebrate taxa. These taxa are listed in order of decreasing contribution to the total dissimilarity between ponds (Table 1).

**Table 1:** SIMPER analysis of macroinvertebrate communities in Bd and non-Bd ponds.

Macroinvertebrate Taxa	Cumulative Contribution	More Abundant In:
*Chironomid sp.*	9.028	Bd ponds
*Physa gyrina*	16.96	non-Bd ponds
*Hesperocorixa spp.*	24.23	Bd ponds
*Chaoborus sp.*	30.88	Bd ponds
*Libellula semifasciata*	34.87	Bd ponds
*Pachydiplax longipennis*	38.7	non-Bd ponds
*Notonecta (juvenile)*	42.3	Bd ponds
*Buenoa spp.*	45.51	Bd ponds
*Dineutus sp.*	48.52	Bd ponds
*Callibaetes sp.*	51.03	Bd ponds

Over 50% of the difference in macroinvertebrate communities between Bd and non-Bd ponds was explained by the abundances of 10 of the 68 total invertebrate taxa (listed in order of decreasing relative contribution).

## Discussion

Despite relatively high levels of incidence and moderate levels of prevalence of Bd in our study area, we found no significant patterns between any of our individual biotic and abiotic descriptors of ponds and presence of Bd in ponds. This is surprising, given findings from other studies of the importance of factors such as temperature [33] on Bd physiology and amphibian density [43] on the presence, prevalence, and intensity of Bd infections in amphibian populations. However, our temperature data for each pond are limited to a single time point, and thus are not expected to be representative of differences in climatic conditions among ponds.

Despite this lack of univariate patterns, we found strong evidence that ponds in which Bd does and does not occur are fundamentally different in terms of their multivariate biotic and abiotic characteristics. These characteristics were consistent of Bd ponds between the conservation areas that we visited, despite the geographical distance between them. The results of our similarity-based ordinations (PERMDISP results and Figs. 2 and 3) suggest that ponds in which we detected Bd are more similar to each other than would be expected based on the diversity of all ponds in our study system. In other words, our results suggest that Bd disproportionately occurs in a nonrandom subset of the pond conditions that are found in our study area. This pattern emerges with respect to amphibian community composition, macroinvertebrate community composition, and physiochemical character of the ponds. Because our data are observational, it is not possible to attribute direct causation among these independent factors, especially because we expect that amphibian and macroinvertebrate community composition and pond physiochemistry interact with each other and potentially with other, unmeasured variables as well.

We used structural equation modeling (SEM) to help address this deficiency. SEMs are used specifically to hypothesize and test causal relationships among correlated variables. Our SEM analysis generated support for a novel and surprising model that suggests that patterns of presence of Bd are best explained by variation in macroinvertebrate community structure, with an indirect effect of pond physiochemistry (Fig. 4). This result is surprising for several reasons. First, the interactions among invertebrates and Bd have generally been ignored and are presumably considered to be less important than well-studied factors such as water temperature and amphibian density (but see [32], [44]). Second, despite the obvious importance of amphibians as the primary host of Bd, our SEM results provide no evidence that variation in amphibian community composition directly explains presence or absence of Bd within a pond. Surprisingly, this result suggests that changes in amphibian diversity and abundance among our study sites were apparently unimportant in determining whether we detected Bd in amphibians in a given pond.

Why would ponds where Bd was found have more similar macroinvertebrate communities than ponds in which Bd did not occur? There are several possible explanations. First, macroinvertebrates species may be directly affecting the probability that *Rana sp.* are infected with Bd by increasing or decreasing the abundance of this pathogen and its infective life stage, the aquatic zoospore. For example, recent research suggests that some invertebrate species may be acting as alternative hosts, which can act as reservoirs or to amplify Bd abundance and infection probability (e.g., crayfish [32]). In contrast they may be reducing zoospore densities via ecological interactions such as competition or predation, as has been documented in microinvertebrate crustaceans (*Daphnia* [44], [45]). As a result, Bd may be limited to the subset of ponds in which alternative hosts occur, or, alternatively, to where important predators of zoospores do not occur.

Alternatively, macroinvertebrate community structure could be responding to some unmeasured underlying biotic or abiotic gradient that also affects presence or absence of Bd. We did not measure dissolved oxygen, dissolved organic matter or ultraviolet light exposure at any of our ponds, and each of these could be relevant to the ecology of Bd [46]. It is also possible that our ponds experienced different levels of anthropogenic disturbance, which is known to correlate with large-scale patterns of Bd occurrence [12], [47]. As our study was exploratory, we had no *a priori* mechanistic hypotheses to explain why presence of Bd and invertebrate community structure might covary. We suggest that the results of our study, combined with recent results showing that some invertebrates can be infected with Bd [32] indicate that more detailed studies on this topic may help explain patterns of distribution of Bd in natural systems.

As a final alternative, it is possible that the presence of Bd causes changes in the structure of the macroinvertebrate community and not vice versa. Although there is some evidence that Bd can infect and cause disease in some invertebrate species [31], [32], there are no published studies on if or how Bd may affect the diversity or structure of invertebrate communities. Although our SEM analysis suggests that Bd incidence responds to invertebrate community structure, because this model is based on correlational data the causal pathway is best interpreted as an hypothesis, and not a conclusion.

A surprising result from our study is that we have no strong evidence that presence or abundance of particular amphibian species significantly affects the distribution of Bd in our study area. This contradicts several past studies, which suggest that host density and diversity can affect pathogen presence, prevalence, and even infection intensity [25], [43], [48]. We did consider the possibility that newts (*N. viridescens*) could be linked to the patterns of macroinvertebrate and amphibian diversity across spatial scales. Owing to their long residence time in ponds and high levels of infection with Bd in the southeastern United States [20], newts are a candidate as a Bd reservoir species and incidence of newts was marginally correlated with incidence of Bd (p = 0.058), having been found in all Bd ponds but only a fraction of non-Bd ponds. Additionally, it is possible that newts, which are important predators in small pond systems [49] contribute to changes in both Bd presence and macroinvertebrate community structure. However, our results do not support this, even when newts were included as an independent factor in our SEM model (not shown). It is also possible that because we focused only on ponds in which *Rana sp.* tadpoles were present, were we unable to detect whether presence of *Rana* affects presence or absence of Bd in ponds.

Our SIMPER analysis was intended to determine whether any individual macroinvertebrate taxon varied drastically in relative abundance between Bd and non-Bd ponds. Macroinvertebrate taxa that contributed largely to the dissimilarity between the communities in Bd and non-Bd ponds could be related to the observed patterns in beta diversity or could be ecologically important for Bd, but because of the large number of species sampled (68), we expect that most or even all of the species we identified are probably over- or underrepresented in Bd ponds due to random chance. How could macroinvertebrate species be involved in the ecology of Bd? Crayfish have been shown to be able to transmit Bd because Bd can infect the keratin lining of their gastrointestinal tracts [32]. Bd has also been experimentally shown to induce mortality in the nematode *C. elegans* by rupturing its cuticle [31], although it is not clear whether Bd can undergo its entire life cycle in a nematode host. However, the possibility that Bd could infect other macroinvertebrate species has remained largely unexplored. Our SIMPER analysis revealed that differences in the abundance of *Chironomid sp.* (midge) larvae between Bd and non-Bd ponds were disproportionately large. *Chironomid sp. larvae* feed on benthic detritus and could encounter Bd zoospores in the sediment; however, it is unknown if or how this could affect presence or abundance of Bd. Likewise, several large macroinvertebrate predators were also more abundant in Bd ponds (*Hesperocorixa spp.*, *Notonecta spp.* juveniles, *Buenoa spp.*, *Libellula semifasciata* and *Dineutus spp.*), but their roles in the ecology of Bd, in mediating amphibian susceptibility, or in structuring macroinvertebrate diversity are unknown. We present these results not as explanations for the patterns we observed, but rather to inspire experimental research exploring how Bd fits into pond food webs or how these species could be involved in patterns of macroinvertebrate diversity across spatial scales.

Our multivariate community-based approach has never been applied to questions about Bd before, and while our study is strictly exploratory, our results do suggest several novel hypotheses. We document clear biological and physiochemical differences between ponds with and without Bd in their amphibian populations. Additionally, we find that patterns of Bd incidence are directly explained by variation in aquatic invertebrate community structure. However, we are only able to speculate on what factors might actually drive this pattern. If researchers are able to identify the ultimate causal factors that best explain presence or absence of this important pathogen within individual habitats, then we may be able to answer the question, “Why does Bd occur in certain amphibian populations, but not others?” The answer to this question could then be used to inform management decisions, identify refuges and amphibian populations at risk, and perhaps to mitigate the negative effects of Bd where it does cause mortality, declines and extinctions.

## Supporting Information

Table S1 **Occupancy of amphibian species in Bd and non-Bd ponds.** Fisher's exact test was used to compare occupancies of each amphibian species between Bd and non-Bd ponds. No amphibian species was found in significantly higher occupancy among Bd or non-Bd ponds (especially after correcting for multiple comparisons), but newts were identified as a potential Bd reservoir.
(DOCX)

Table S2 **Univariate abiotic correlates of Bd incidence.** Logistic regressions were performed to assess whether any environmental variables correlated with the incidence of Bd. No significant results were found.
(DOCX)

Table S3 **Univariate biotic correlates of Bd incidence.** Logistic regressions were performed to assess whether any environmental variables correlated with the incidence of Bd. No significant results were found.
(DOCX)
