# Supplementary material for: Why Does Amphibian Chytrid (Batrachochytrium dendrobatidis) Not Occur Everywhere? An Exploratory Study in Missouri Ponds
Source: PLoS One. 2013 Sep 25;8(9):e76035. doi: 10.1371/journal.pone.0076035 (PMC3783386; doi:10.1371/journal.pone.0076035)
Supplement: Table S1 — Occupancy of amphibian species in Bd and non-Bd ponds. Fisher's exact test was used to compare occupancies of each amphibian species between Bd and non-Bd ponds. No amphibian species was found in significantly higher occupancy among Bd or non-Bd ponds (especially after correcting for multiple comparisons), but newts were identified as a potential Bd reservoir. (DOCX) [file pone.0076035.s001.docx]

| **Amphibian Species** | **Odds ratio** | **95% C.I.** | **p value (α=.005)** |
| --- | --- | --- | --- |
| Central Newt (*N. viridescens*) | 0.000 | 0.000; 1.206 | 0.058 |
| Blanchards’s Cricket Frog (*A. crepitans*) | ∞ | 0.421; ∞ | 0.268 |
| Gray Treefrog (*H. versicolor/chrysoscelis*) | 3.692 | 0.332; 199.6 | 0.362 |
| Green Frog (*R. clamitans*) | 0.600 | 0.076; 3.768 | 0.694 |
| Spring Peeper (*P. crucifer*) | 0.683 | 0.104; 4.645 | 0.694 |
| Southern Leopard Frog (*R. sphenocephala*) | 1.384 | 0.239; 8.936 | 0.717 |
| Spotted Salamander (*A. maculatum*) | 1.107 | 0.078; 11.75 | 1 |
| American Toad (*B. americanus*) | ∞ | 0.016; ∞ | 1 |
| Pickerel Frog (*R. pulustris*) | ∞ | 0.016; ∞ | 1 |
