# Supplementary material for: Why Does Amphibian Chytrid (Batrachochytrium dendrobatidis) Not Occur Everywhere? An Exploratory Study in Missouri Ponds
Source: PLoS One. 2013 Sep 25;8(9):e76035. doi: 10.1371/journal.pone.0076035 (PMC3783386; doi:10.1371/journal.pone.0076035)
Supplement: Table S2 — Univariate abiotic correlates of Bd incidence. Logistic regressions were performed to assess whether any environmental variables correlated with the incidence of Bd. No significant results were found. (DOCX) [file pone.0076035.s002.docx]

| **Abiotic Predictor** | **Odds ratio** | **95% C.I.** | **Estimate** | **p value** |
| --- | --- | --- | --- | --- |
| Sampling Date | 0.939 | 0.833; 1.058 | -0.063 | 0.302 |
| Water Temperature | 1.177 | 0.918; 1.511 | 0.163 | 0.199 |
| Nearest Neighbor Pond Distance† | 1.785 | 0.864; 3.686 | 0.579 | 0.117 |
| Conductivity† | 0.000 | 0.000; 594.3 | -28.058 | 0.110 |
| pH | 0.193 | 0.026; 1.444 | -1.644 | 0.109 |
| Total Nitrogen† | 0.316 | 0.074; 1.349 | -1.152 | 0.120 |
| Total Phosphorus† | 0.050 | 0.001; 2.153 | -2.988 | 0.119 |
| † data were log transformed to improve normality | | | | |
