# Supplementary material for: Why Does Amphibian Chytrid (Batrachochytrium dendrobatidis) Not Occur Everywhere? An Exploratory Study in Missouri Ponds
Source: PLoS One. 2013 Sep 25;8(9):e76035. doi: 10.1371/journal.pone.0076035 (PMC3783386; doi:10.1371/journal.pone.0076035)
Supplement: Table S3 — Univariate biotic correlates of Bd incidence. Logistic regressions were performed to assess whether any environmental variables correlated with the incidence of Bd. No significant results were found. (DOCX) [file pone.0076035.s003.docx]

| **Biotic Predictor** | **Odds ratio** | **95% C.I.** | **Estimate** | **p value** |
| --- | --- | --- | --- | --- |
| Chlorophyll-a† | 0.167 | 0.011; 2.513 | -1.787 | 0.196 |
| Total Amphibian Density† | 0.854 | 0.439; 1.658 | -0.158 | 0.641 |
| *Rana sp.* Density† | 0.856 | 0.470; 1.560 | -0.155 | 0.612 |
| *N. viridescens* Density† | 1.202 | 0.564; 2.561 | 0.184 | 0.634 |
| Total Zooplankton Density† | 0.616 | 0.265; 1.433 | -0.485 | 0.260 |
| Simpson’s Diversity (Amphibians) | 0.088 | 0.001; 5.170 | -2.430 | 0.242 |
| Simpson’s Diversity (Invertebrates) | 51.844 | 0,583; 4,612.0 | 3.948 | 0.085 |
| Simpson’s Diversity (Zooplankton) | 0.638 | 0.001; 532.58 | -0.450 | 0.896 |
| † Indicates data were log transformed to improve normality | | | | |
